# Isolated Ventricular Inversion: A Rare Complex Congenital Heart Disease With Neonatal Cyanosis

**DOI:** 10.1016/j.jaccas.2022.09.012

**Published:** 2022-11-03

**Authors:** Arthur Gavotto, Marie Vincenti, Sophie Guillaumont

**Affiliations:** a Pediatric and Congenital Cardiology Department, M3C Regional Center, University Hospital, Montpellier, France; b PhyMedExp, University of Montpellier, Centre National de la Recherche Scientifique, Institut National de la Santé et de la Recherche Médicale, Montpellier, France

**Keywords:** atrioventricular discordance, congenital heart disease, isolated ventricular inversion, pediatric, VSD, ventricular septal defect

## Abstract

A 1-day-old girl was referred for a cardiology consultation for a mean saturation at 80% without respiratory distress. Echocardiography showed an isolated ventricular inversion. This entity is extremely rare, with fewer than 20 cases reported. This case report describes the clinical evolution and the complex surgical management of this pathology. (**Level of Difficulty: Advanced.**)

## Central Illustration

**Figure:**
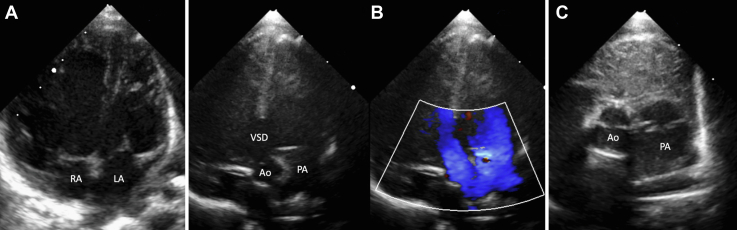

## Case Presentation

**Figure 1:**
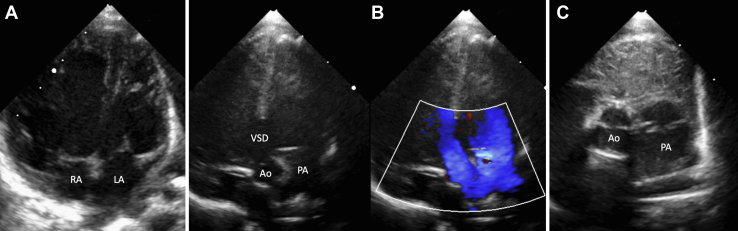
Echocardiography **(A)** The apical 4-chamber view. **(B)** The anterior apical 5-chamber view with and without color Doppler. **(C)** The parasternal short-axis view focused on great arteries. Ao = aorta; LA = left atrium; PA = pulmonary artery; RA = right atrium; VSD = ventricular septal defect.

A 1-day-old girl was referred for a cardiology consultation following a prenatal diagnosis of complex congenital heart disease. Vaginal delivery occurred at 39 weeks, and the newborn presented a good adaptation to extrauterine life. The mean saturation was 80% with spontaneous breathing in ambient air and no respiratory distress. Physical examination demonstrated a grade II holosystolic murmur and no hepatomegaly. Echocardiography showed a levocardia, a right atrium in anatomic position receiving 2 vena cava, and a left atrium in anatomic position receiving 4 pulmonary veins. A 3-mm patent foramen ovale and a large 10-mm muscular ventricular septal defect (VSD) extending to the conal septum shunted bidirectionally. Regarding the great vessels, the left aortic arch with isthmic hypoplasia and normal-sized pulmonary artery were linked by a large ductus arteriosus (bidirectional shunt). Figure 1 shows the apical 4-chamber view (Figure 1A), the anterior apical 5-chamber view to see the ventriculoarterial connection with and without color Doppler (Figure 1B), and the parasternal short-axis view focused on the great arteries (Figure 1C).

## Discussion

The diagnosis of this congenital heart disease is an atrioventricular discordance with a concordant ventriculoarterial connection. The echocardiography showed a morphologic right ventricle in the left anatomic position and a morphologic left ventricle in the right anatomic position. The great arteries demonstrated a parallel course and a ventriculoarterial concordance with the aorta essentially filled by the left ventricle. The segmental analysis concluded to {situs solitus viscero atrial, L-loop, solitus normal great arteries}, where the infundibulum had a superoinferior orientation rather than right to left in {situs solitus viscero atrial, D-loop, solitus normal great arteries}, giving the appearance of parallel great arteries in the front view and crossed in the side view. The high-detail anatomic description of atrioventricular discordance with VSD, ventriculoarterial concordance, and aortic isthmus hypoplasia was made using transthoracic echocardiography from the prenatal period and confirmed by postnatal echography. We completed the postnatal evaluation by computed tomography scan only to confirm the coronary artery anatomy and help the surgeon to plan the patient’s surgery as precisely as possible.

The combination of a discordant atrioventricular connection with a concordant ventriculoarterial connection is also called “isolated ventricular inversion.”^1^ Described for the first time by Van Praagh and Van Praagh^2^ in 1966, this entity is an extremely rare congenital heart disease; Ranjit et al^3^ reviewed 15 descriptions with usual atrial arrangement in 1991, and the Parisian team reported 8 cases essentially associated with heterotaxy syndrome in a 30-year study period.^4^

The physiology of this cardiac anomaly is similar to transposition of the great arteries. The neonatal evolution is marked by heart failure secondary to increased pulmonary blood flow with pulmonary arterial hypertension. A banding of the 2 pulmonary arteries with preservation of the ductus arteriosus was done at 3 months of life. The evolution was marked by a persistence of heart failure and cyanosis as well as the apparition of a right ventriculoarterial valve regurgitation (eg, mitral valve) on prolapsus. Complete corrective surgery was performed at 11 months of life: aortic arch reconstruction with suture section of the ductus arteriosus under hypothermia and anterograde perfusion of brachiocephalic trunk, followed by a standard cardiopulmonary bypass for closure of the VSD via a right atriotomy, the mitral valve plasty, and realization of the Senning procedure. The surgery was complicated by complete atrioventricular block requiring the placement of an epicardial pacemaker 9 days later.

## Funding Support and Author Disclosures

The authors have reported that they have no relationships relevant to the contents of this paper to disclose.
